# Molecular insights into the functional mechanism of phosphoserine phosphatase SerB2 in *Mycobacterium tuberculosis*

**DOI:** 10.3389/fmicb.2026.1863671

**Published:** 2026-07-02

**Authors:** Dafeng Liu, Huashui Deng, Xinbao Liu, Wenshuang Yao, Na Li

**Affiliations:** 1 Key Laboratory of Microbial Resources Protection, Development and Utilization, College of Biological Sciences and Technology, Yili Normal University, Yining, Xinjiang, China; 2 School of Life Sciences, Xiamen University, Xiamen, Fujian, China

**Keywords:** gene expression levels, molecular docking, molecular dynamics simulation, *Mycobacterium tuberculosis*, phosphoserine phosphatase SerB2

## Abstract

*Mycobacterium tuberculosis* (*Mtb*) phosphoserine phosphatase SerB2 is a key enzyme essential for bacterial survival and virulence, catalyzing the final step in the L-serine biosynthesis pathway. SerB2 is an attractive drug target due to its role in immune evasion by *Mtb*. However, the functional mechanism of *Mtb* SerB2 remains poorly understood. Here, we measured the hydrodynamic radius of monomeric SerB2 to be 5.4 ± 0.3 nm. Based on an AlphaFold2-predicted structural model, molecular docking was performed and site-specific mutations were carried out. Mutants D185A, D187A, E194A, S226A, R230A or K318A exhibited significantly reduced activity. In contrast, deletion of segment 294–314 (Δ294–314) increased activity. Notably, the 294–314 region exhibited higher flexibility compared to other parts of SerB2. Among the inhibitors tested, clofazimine displayed the most potent inhibitory effect. Our findings offer new insights into the mechanisms of SerB2 function and lay the structural and biochemical groundwork for developing novel anti-tuberculosis therapies.

## 1 Introduction

Tuberculosis (TB), caused by *Mycobacterium tuberculosis* (*Mtb*), remains a major global health threat, with significant implications for public safety (Trajman et al., 2025; Schmidt et al., 2025). Despite decades of intensive chemotherapy, approximately one-quarter of the world’s population is still infected with *Mtb* (World Health Organization).^1^ TB is further exacerbated by co-morbidities, including HIV and SARS-CoV-2 infections (Dheda et al., 2022; Flores-Lovon et al., 2022; Udoakang et al., 2023; Dhana et al., 2022; Rule et al., 2021). The situation is increasingly dire due to the rise and spread of *Mtb* strains resistant to key drugs, such as isoniazid, pyrazinamide, and rifampicin (Dartois et al., 2025; Farhat et al., 2024; Dheda et al., 2024; Goossens et al., 2021; Liu et al., 2025a; Liu et al., 2025d). These drug-resistant strains pose a major challenge to effective treatment, resulting in high mortality rates. In light of these challenges, it is imperative to return to the fundamentals of drug discovery, focusing on identifying new therapeutic targets and developing novel drug candidates.

*Mtb* phosphoserine phosphatase SerB2 has been identified as a potential therapeutic target through transposon mutagenesis aimed at identifying genes essential for pathogen growth (Sassetti et al., 2003; Arora et al., 2014; Mande et al., 2014). *Mtb* SerB2 can be secreted into macrophages, where it dephosphorylates NF-κB and MAPK-p38, key enzymes involved in inflammation and immune response (Shree et al., 2016; Haufroid and Wouters, 2019). Additionally, SerB2 activates cofilin through dephosphorylation, leading to rearrangement of the macrophage cytoskeleton. Inhibition of SerB2 results in the reactivation of the host immune system (Pierson et al., 2020; Pierson et al., 2023). SerB2 catalyzes the dephosphorylation of O-phospho-L-serine to L-serine (Shree et al., 2016; Haufroid and Wouters, 2019; Pierson et al., 2020; Pierson et al., 2023). Structurally, SerB2 consists of three domains: two ACT-type regulatory domains, which facilitate retroinhibition of the dephosphorylation reaction through binding of serine (the product of the reaction) to the ACT1 domain, and a phosphoserine phosphatase domain that catalyzes the dephosphorylation reaction. The latter domain contains conserved motifs typical of enzymes in this family. However, the relationship between the structure and functional mechanism of SerB2 remains poorly understood.

Herein, the hydrodynamic radius of monomeric SerB2 was determined to be 5.4 ± 0.3 nm. Molecular docking was performed using an AlphaFold2-predicted structural model, and site-specific mutations were introduced. Mutants D185A, D187A, E194A, S226A, R230A, or K318A exhibited significantly reduced activity. In contrast, the deletion of 294–314 segment (Δ294–314) enhanced enzyme activity. Notably, the 294–314 segment showed greater flexibility than other regions of SerB2. Clofazimine exhibited the most potent inhibitory activity among all tested inhibitors in this study. These results clarify the role of SerB2 in the mechanism of action and provide a structural and biochemical basis for the development of new antitubercular agents.

## 2 Results

### 2.1 Characterization of SerB2 using dynamic light scattering

**FIGURE 1:**
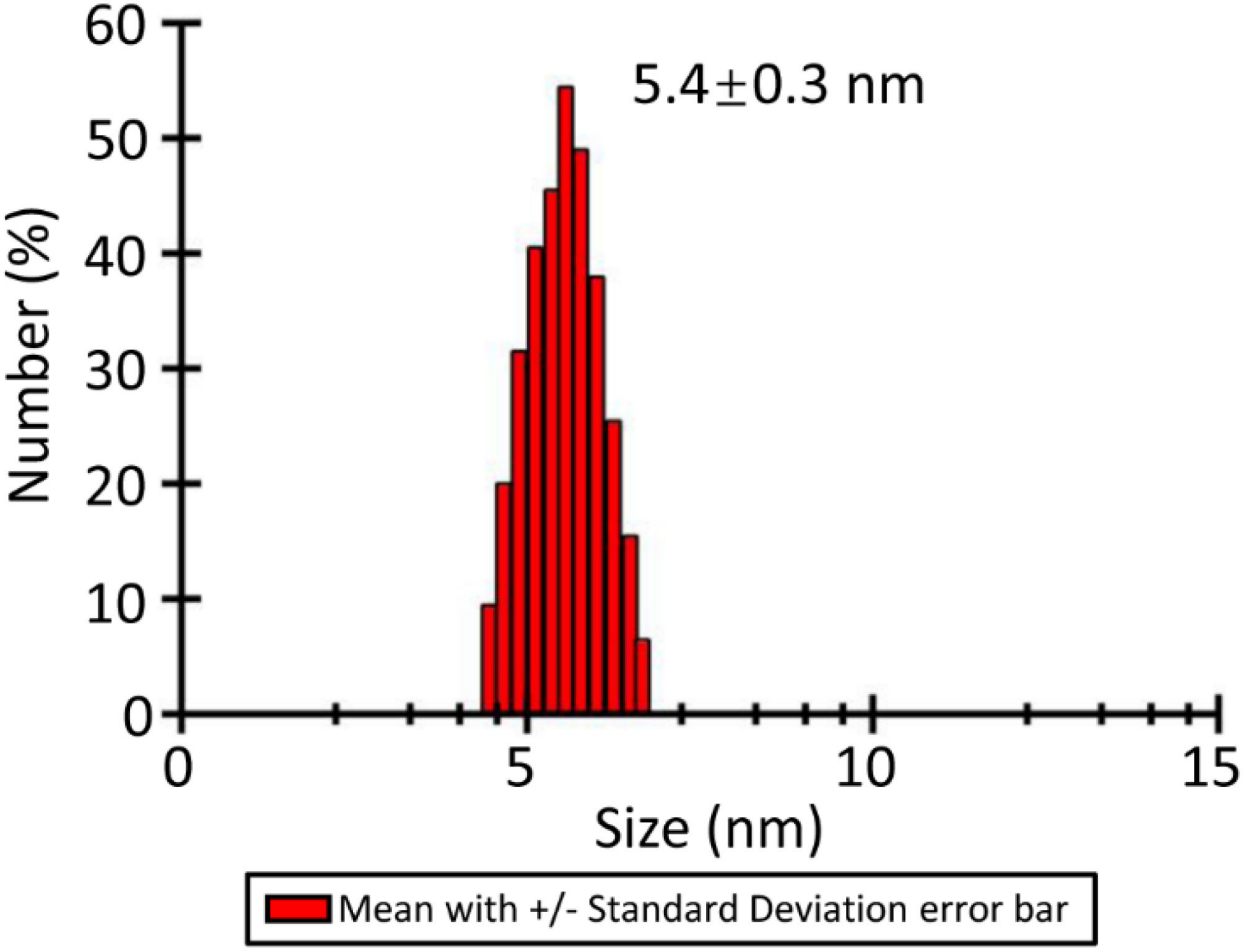
The DLS (Dynamic light scattering) spectrum of SerB2. The hydrodynamic radius of SerB2 was determined to be 5.4 ± 0.3 nm.

The oligomeric status of SerB2 was assessed by dynamic light scattering (DLS) following centrifugation to determine its hydrodynamic radius. The measurements yielded a hydrodynamic radius of 5.4 ± 0.3 nm (Figure 1), suggesting that SerB2 might be a monomeric form.

### 2.2 Prediction and validation of SerB2 structural model

The three-dimensional (3D) structure of SerB2 was predicted using AlphaFold2 (Wayment-Steele et al., 2023; Jumper et al., 2021). AlphaFold2 uses deep learning algorithms for more accurate and reliable protein structure predictions compared to traditional homology modeling methods.

**FIGURE 2:**
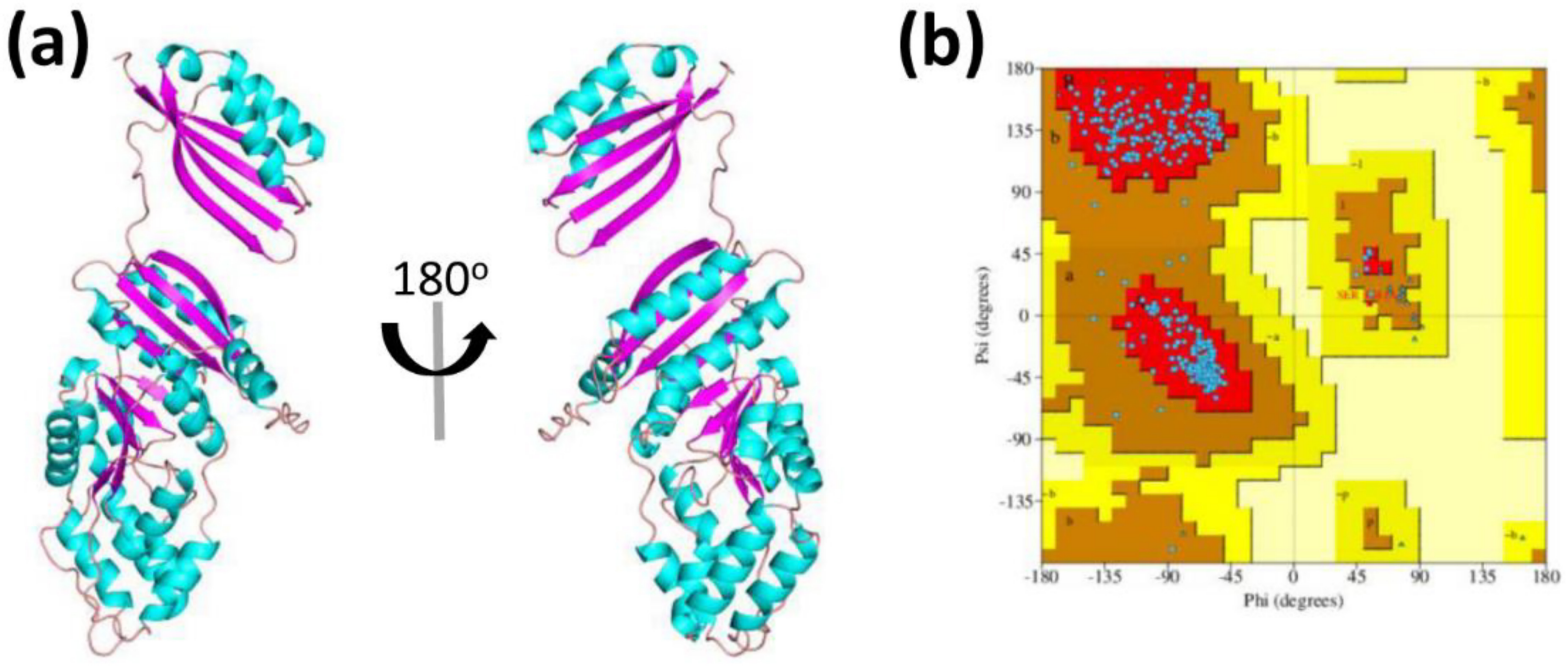
Prediction and evaluation of structural model of SerB2. **(a)** The structural model, predicted using AlphaFold2, is depicted from two perspectives in a ribbon diagram, where alpha-helices are colored cyan and beta-sheets are pink. **(b)** Validation of the structural model by Ramachandran plot analysis indicated favorable stereochemical quality, with the most favored regions indicated in red and the less favored regions in progressively lighter shades.

To evaluate the quality of the predicted SerB2 model (Figure 2a and Supplementary Figure S1), a Ramachandran plot was used to assess the dihedral angles of the protein backbone and verify their placement within acceptable regions, indicating a reasonable conformation. The results revealed that 94.8% of residues were in the most favored region, 4.9% in the additionally allowed region, 0.3% in the generously allowed region, and none in the disallowed region (Figure 2b, Supplementary Figure S1 and Table 1). The structure also contained 2 terminal residues, 41 glycine residues, and 19 proline residues (Figure 2b, Supplementary Figure S1, and Table 1). With over 90% of residues in the most favored regions, these results show that the predicted model is of high quality.

**TABLE 1:** Ramachandran plot statistics of structural models of SerB2.

Residual properties	Number of residues^b^	Total % of residues
Most favored region^a^	329	94.8
Additional allowed region	17	4.9
Generously allowed region	1	0.3
Disallowed region	0	0

^a^A good quality model is expected to have over 90% residues in most favored regions (Chen et al., 2009).

^b^Number of end-residues (excl. Gly and Pro): 2; ^b^Number of glycine residues (shown as triangles): 41; ^b^Number of proline residues: 19.

Overall, the Ramachandran plot assessment revealed that 94.8% of residues occupy the most favored region, exceeding the 90% threshold and demonstrating excellent model quality. Therefore, the predicted model is suitable for further study.

### 2.3 Predicted binding site residues of SerB2 with O-Phospho-L-Serine

**FIGURE 3:**
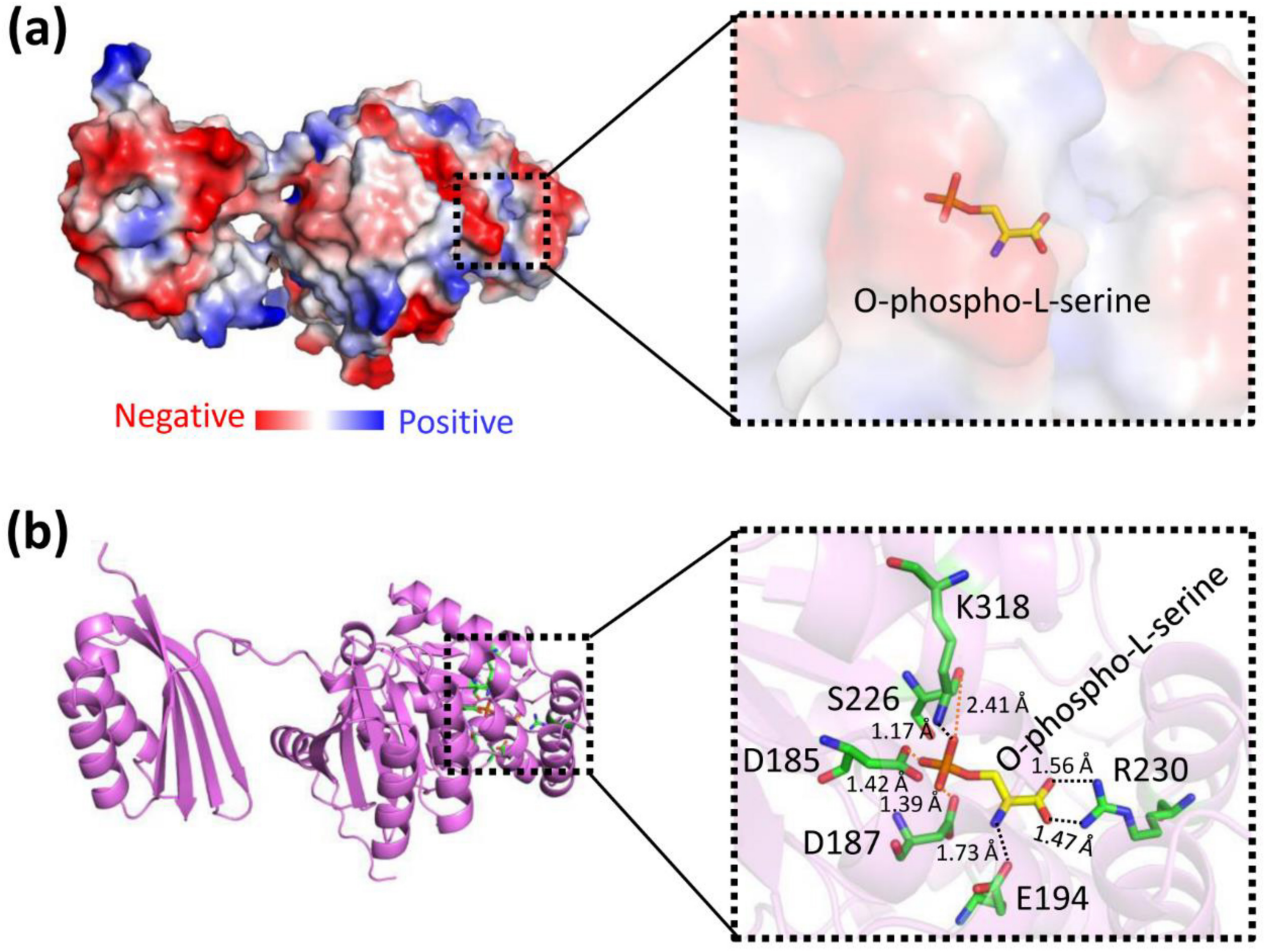
The structural model of the binary complex of SerB2 and O-phospho-L-serine. A stick model of the substrate O-phospho-L-serine is shown. **(a)** The surface electrostatic potential of SerB2 is depicted, with negative, positive, and neutral regions colored red, blue, and white, respectively. **(b)** A ribbon representation features an inset highlighting the binding pocket, where key residues (D185, D187, E194, S226, R230, K318) engage in electrostatic interactions, indicated by dashed lines (on the right).

Molecular docking was performed using AutoDock software (Morris et al., 2009; Trott and Olson, 2009; Forli et al., 2016) to model the complex between SerB2 and its substrate, O-phospho-L-serine, based on the predicted structural model. The binding energy of −5.92 kcal/mol indicates that the results may be reliable. In the protein-substrate complex model, O-phospho-L-serine was effectively accommodated in SerB2 binding pocket, which possessed negative charges (Figure 3a). Key residues within the pocket (D185, D187, E194, S226, R230, and K318) interact with O-phospho-L-serine through hydrogen bonds and electrostatic interactions (Figure 3b).

### 2.4 Enzymatic activity assays of SerB2 and its mutant variants

**FIGURE 4:**
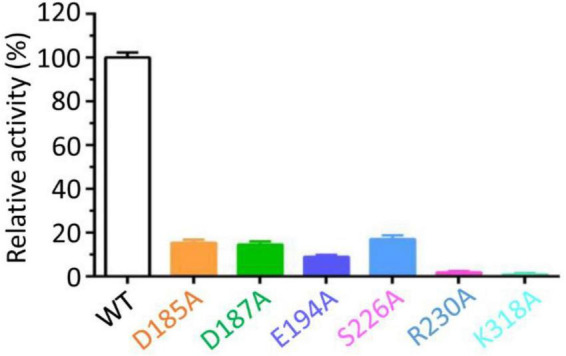
Enzymatic activity of WT (wild-type) SerB2 and its mutants. These mutations (D185A, D187A, E194A, S226A, R230A, and K318A) significantly decreased SerB2 activity, reducing it by 5–120-fold compared to WT (wild-type) SerB2.

Based on the molecular docking results (Figure 3), site-specific mutations were introduced. Mutants D185A, D187A, E194A, S226A, R230A, or K318A exhibited a 6- to 120-fold decrease in activity compared to the wild-type (WT) protein (Figure 4). These findings indicated the crucial role of residues D185, D187, E194, S226, R230, and K318 in the catalytic activity of SerB2.

### 2.5 Deletion of segment 294–314 (Δ294–314) significantly increased the activity

**FIGURE 5:**
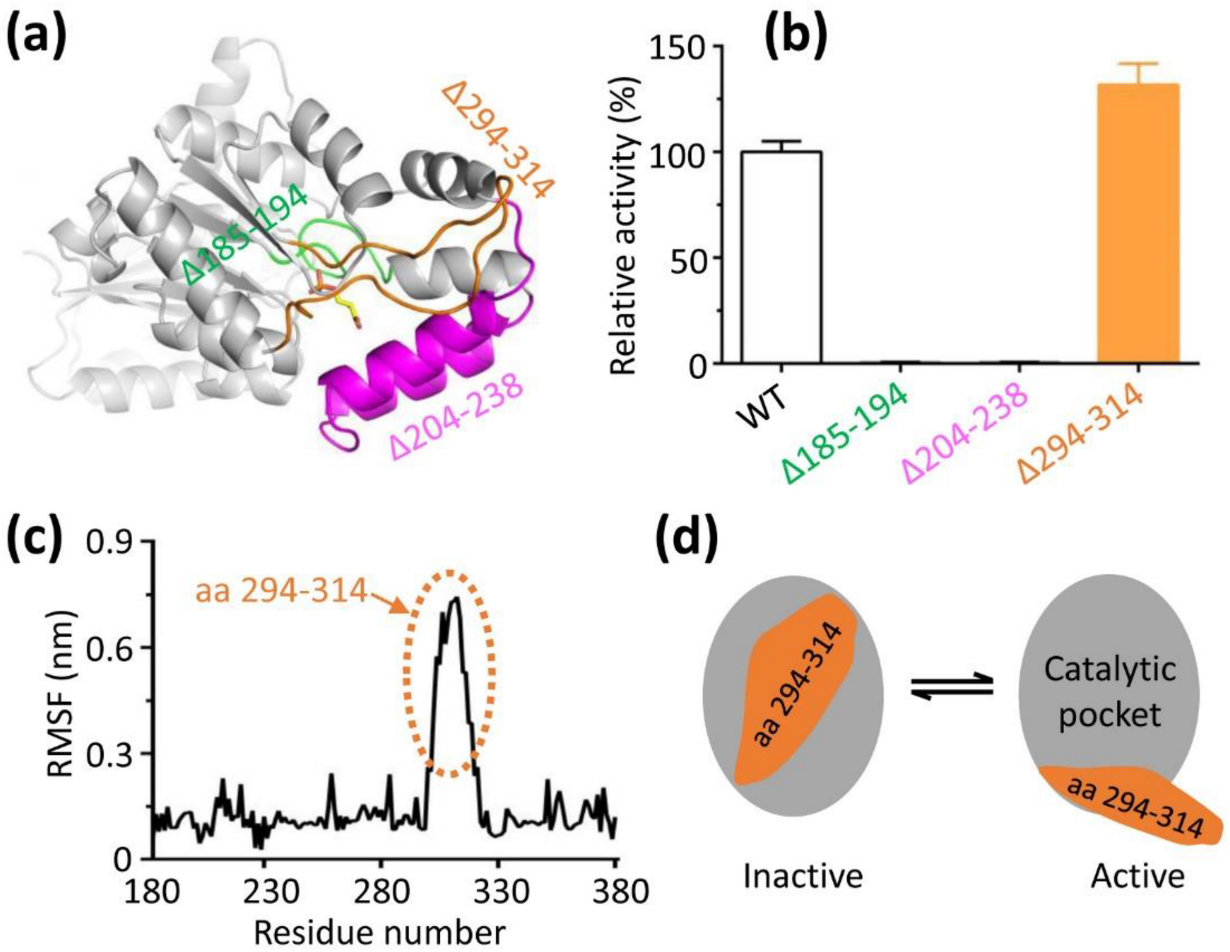
Molecular regulation of SerB2 activity. **(a)** The segments comprising residues 185–194, 204–238, and 294–314 are colored green, magenta, and orange, respectively. **(b)** Deletions of 185–194 or 204–238 (Δ185–194 or Δ204–238) led to a complete loss of activity. Conversely, the deletion of 294–314 (Δ294–314) enhanced activity. The activity of WT SerB2 was set to 100%. **(c)** The high flexibility of segment 294–314 based on the results of RMSF (root mean square fluctuation) profile of SerB2. The region 294–314 of high volatility was identified, as evidenced by a pronounced peak in RMSF profile. RMSF quantifies local flexibility by measuring the average deviation of backbone atom positions over the simulation trajectory. **(d)** Catalytic activity of SerB2 is modulated by the dynamic flexibility of the 294–314 segment. Structural enlargement of the catalytic pocket, resulting from the rearrangement of the segment (residues 294–314, in orange), facilitates substrate binding and consequently activate the catalytic activity of SerB2.

Regions 185–194, 204–238, and 294–314 are located around the periphery of SerB2 catalytic pocket (Figure 5a). Deletions of regions 185–194 or 204–238 (Δ185–194 or Δ204–238) resulted in a complete loss of activity (Figure 5b), implying the critical role of these regions in SerB2 catalytic function. In contrast, deletion of the 294–314 region (Δ294–314) significantly enhanced activity compared to the wild-type (WT) protein (Figure 5b). This suggests that the 294–314 region undergoes conformational changes that impede the binding of the substrate (O-phospho-L-serine) to the catalytic pocket, thus inhibiting the catalytic reaction. These findings indicate that the flexibility of the 294–314 region is important for modulating SerB2 activity.

### 2.6 The dynamic flexibility of segment 294–314 modulates catalytic activity

To investigate the flexibility of segment 294–314, we performed molecular dynamics simulations. The flexibility of each region of SerB2 was assessed using root mean square fluctuation (RMSF), which quantifies atomic displacement relative to the average position. The RMSF analysis revealed that segment 294–314 exhibited a peak fluctuation of 0.73 nm, significantly higher than other regions of SerB2 (Figure 5c), confirming its high flexibility.

Based on these structural and functional insights, we propose that conformational flexibility of residues 294–314 modulates catalytic activity of SerB2 (Figure 5d). SerB2 exists in an equilibrium between active and inactive states in solution. Segment 294–314 regulates the interaction between SerB2 and its substrate (O-phospho-L-serine). When the catalytic pocket contracts, preventing substrate binding and rendering SerB2 inactive. In contrast, the catalytic pocket expands, facilitating substrate binding and activating the enzyme. This structural arrangement positions SerB2 and its substrate for efficient catalysis.

### 2.7 Effect of the inhibitor on SerB2 activity

**FIGURE 6:**
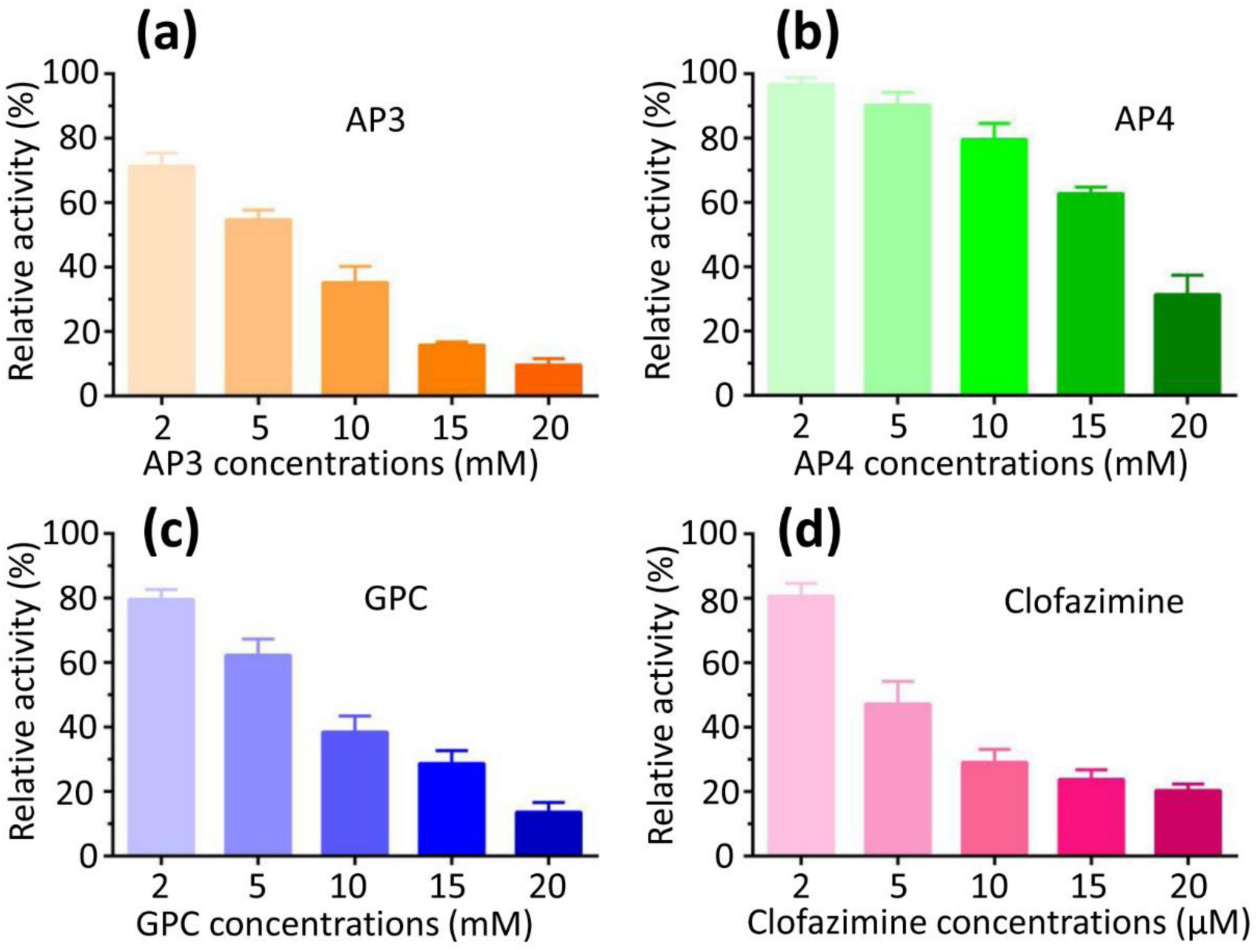
Inhibition of SerB2 activity on addition of increasing concentration of inhibitors. The SerB2 shows over 50% reduction in activity when inhibitors **(a)** AP3 (2-amino-3-phosphonopropionic acid), **(b)** AP4 (2-amino-4-phosphonobutyric acid), **(c)** GPC (L-alpha-glycerophosphorylcholine), and **(d)** clofazimine were at 10, 20, 10 mM, and 5 μM, respectively.

SerB2 is a key enzyme in the L-serine biosynthetic pathway and is essential for *Mtb* viability (Sassetti et al., 2003; Shree et al., 2016). Previous studies have identified SerB2 as a promising drug target (Mande et al., 2014; Arora et al., 2014; Shree et al., 2016). To assess the inhibitory effects of compounds AP3 (2-amino-3-phosphonopropionic acid), AP4 (2-amino-4-phosphonobutyric acid) (Hawkinson et al., 1997), GPC (L-alpha-glycerophosphorylcholine) (Hawkinson et al., 1997), and clofazimine (Sassetti et al., 2003; Cholo et al., 2012; Arora et al., 2014; Mande et al., 2014; Shree et al., 2016), we measured SerB2 activity in the presence of varying concentrations of each inhibitor. We found that AP3, AP4, GPC, and clofazimine inhibited SerB2 by over 50% at concentrations of 10, 20, 10, and 5 μM, respectively (Figures 6a–d). These results are consistent with the previously reported IC_50_ of approximately 5.0 μM, *K*_*i*_ value of 2.7 μM, and *K*_*m*_ value of 135.9 μM for the inhibitor clofazimine (Shree et al., 2016). Notably, clofazimine exhibited the strongest inhibitory effect among these inhibitors. Based on these results, clofazimine was selected for further investigation in subsequent experiments.

### 2.8 Effects of 10 μM clofazimine on the activity of SerB2 and its mutant variants

**FIGURE 7:**
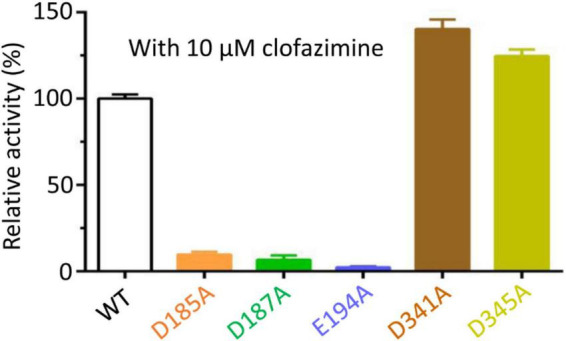
Enzymatic activity of WT SerB2 and mutants with 10 μM clofazimine. D185A, D187A, or E194A significantly decreased activity. However, D341A or D345A dramatically increased the activity. The activity of WT (wild-type) protein was set to 100% at 10 μM clofazimine.

Based on the results of SerB2 inhibition by increasing inhibitor concentrations (Figure 6), we performed site-specific mutagenesis experiments. D185, D187, and E194 were selected for mutation based on molecular docking results (Figure 3), while D341 and D345 were chosen based on previous studies (Shree et al., 2016). Mutations D185A, D187A, or E194A resulted in a significant decrease in activity at 10 μM clofazimine compared to the wild-type (WT) protein (Figure 7). In contrast, mutations D341A and D345A led to a significant increase in activity at the same inhibitor concentration (Figure 7).

Residues D185, D187, and E194 are likely critical for the catalytic reaction or maintaining the active conformation of SerB2. Mutating these residues to glycine (D185A, D187A, E194A) disrupts their function, leading to reduced activity. Conversely, the residues D341 and D345 may play a role in the binding of clofazimine or the allosteric regulation of SerB2. Mutations at these sites (D341A and D345A) may impair inhibitor binding or induce a conformational shift toward an active state. This results in increased activity compared to the wild-type (WT) protein in the presence of clofazimine.

### 2.9 Expression profiles of *SerB2* using RT-qPCR

**FIGURE 8:**
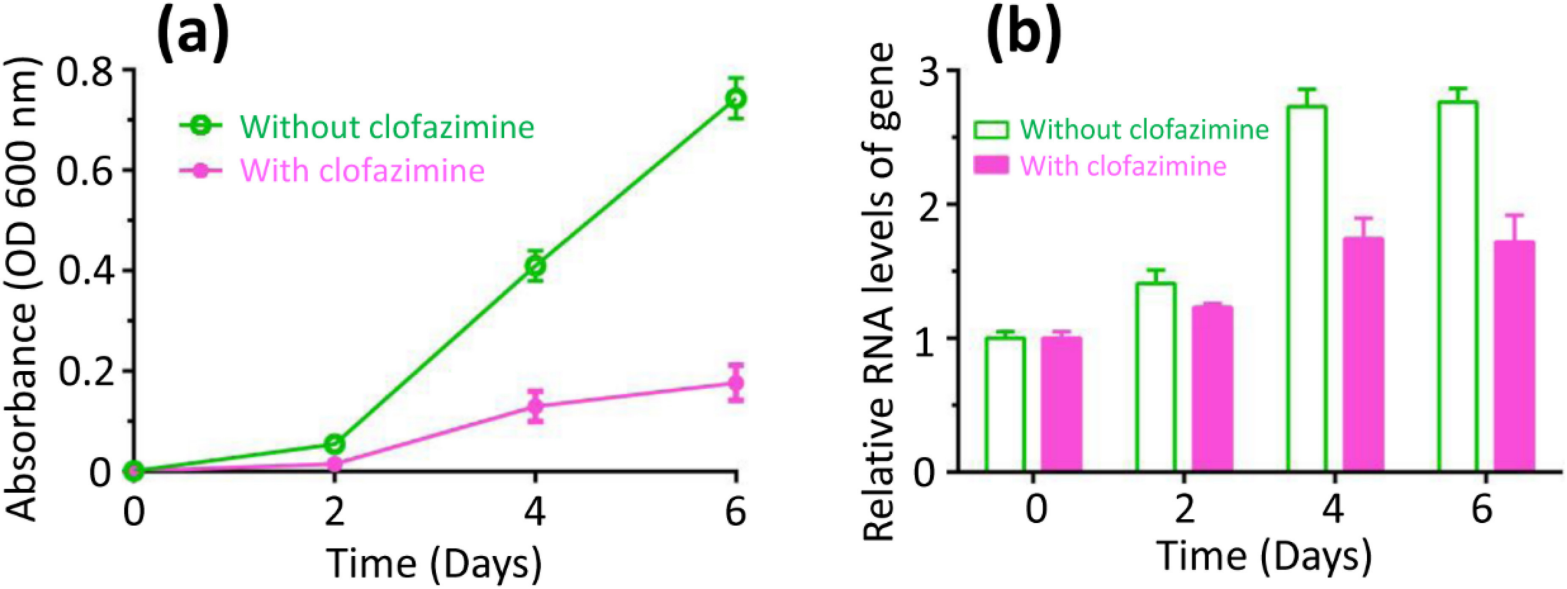
Functional role of SerB2. **(a)** Growth curves of bacterial strains treated with 0 or 10 μM clofazimine. **(b)** The expression levels of *SerB2* gene were assessed at various time points when bacterial strains were treated with 0 or 10 μM clofazimine. The resulting data are presented as log_2_-fold changes using RT-qPCR (reverse transcription quantitative PCR), where positive values signify transcriptional up-regulation.

Growth curve analysis of *Mtb* strains treated with clofazimine indicated an inhibitory effect on growth (Figure 8a), suggesting that *SerB2* gene may be essential for *Mtb* survival. To examine the expression profile of *SerB2*, RT-qPCR (real-time quantitative polymerase chain reaction) was performed at various time points with bacterial strains treated with either 0 or 10 μM clofazimine. *SerB2* transcription levels increased progressively over time (Figure 8b). Specifically, without clofazimine treatment, *SerB2* expression was upregulated by 1. 4-, 2. 7-, and 2.8-fold on days 2, 4, and 6, respectively. With clofazimine treatment, *SerB2* expression was upregulated by 1. 2-, 1. 7-, and 1.7-fold on the same days (Figure 8b). These findings reinforce that SerB2 is crucial for Mtb survival, consistent with previous reports (Arora et al., 2014; Mande et al., 2014; Shree et al., 2016; Grant, 2017; Pierson and Wouters, 2020; Haufroid et al., 2023; Pierson et al., 2023).

## 3 Discussion

In this work, the hydrodynamic radius of monomeric SerB2 was measured as 5.4 ± 0.3 nm. Molecular docking, based on an AlphaFold2-predicted structural model, was performed alongside site-specific mutagenesis. Mutants D185A, D187A, E194A, S226A, R230A, and K318A exhibited a dramatic decrease in activity, while deletion of the 294–314 segment (Δ294–314) resulted in enhanced activity. The 294–314 region showed greater flexibility compared to other regions of SerB2. Among the inhibitors tested, clofazimine exhibited the strongest inhibitory effect. These results elucidate the molecular mechanism of SerB2 and offer an essential structural and biochemical basis for the rational design of novel anti-tuberculosis agents.

The structural interpretations presented in this study are predominantly based on a model predicted using AlphaFold2. Although this model offers a useful framework for generating hypotheses, it remains a computational prediction that has not been corroborated by high-resolution experimental data. Consequently, the suggested contributions of specific residues to substrate binding, transition-state stabilization and catalysis should be considered preliminary until validated using experimental structural methods such as X-ray crystallography or cryo-electron microscopy. While our mutagenesis and biochemical data strongly indicate the functional significance of these residues, the detailed molecular mechanisms deduced from the model are still conjectural. These have motivated our ongoing efforts to experimentally determine the three-dimensional structure of SerB2 using X-ray crystallography or other methods.

The AlphaFold2-predicted model of SerB2 adopts a canonical haloacid dehalogenase (HAD) superfamily fold with a core Rossmannoid domain. Notably, the model accurately positions key residues D185, D187, and E194 within the active site, forming a conserved catalytic triad. Our experimental data strongly validate this computational prediction: mutations D185A, D187A, and E194A each led to a significant loss of enzymatic activity. These results confirm that D185 likely serves as the nucleophile, attacking the phosphorous atom of the substrate, O-phospho-L-serine, to form a phosphoaspartate intermediate. The complete inactivation of these mutants underscores their essential roles in the catalytic mechanism, bridging the gap between *in silico* predictions and experimental biochemistry.

In the molecular docking, docking scores are semi-empirical estimates from simplified scoring functions and therefore cannot independently validate binding accuracy or biological relevance. Although a favorable score indicates a thermodynamically plausible binding pose, it does not account for protein flexibility, explicit solvent effects, or entropic contributions, nor does it guarantee that the predicted pose represents the biologically active conformation. Accordingly, the docking energy is presented solely as an indication of a potentially favorable binding mode and should be interpreted cautiously, together with experimental data.

To further explore the structural and functional basis of SerB2 catalysis, we are investigating its crystal structure using X-ray crystallography.

Although Clofazimine is known to have multiple targets, our findings uncover a novel, specific interaction with this critical metabolic enzyme. Molecular docking, based on our AlphaFold2 model, suggests that Clofazimine binds within the active site, potentially competing with the substrate or disrupting the catalytic machinery. This not only provides new mechanistic insights into Clofazimine’s anti-tubercular activity but also demonstrates that the SerB2 active site is a viable drug target. The structure-activity relationships derived from our mutagenesis studies further guide the design of more potent and selective inhibitors targeting this site.

Several limitations of this study should be carefully considered.

(1) The three-dimensional structure of the target protein SerB2 was derived from AlphaFold2 prediction. Although AlphaFold2 provides models with high per-residue confidence, it remains a computational prediction and may not fully capture ligand-induced conformational changes, flexible loop dynamics, or the subtle side-chain rearrangements that occur upon binding. Consequently, the docked poses and the inferred interaction patterns must be regarded as hypotheses rather than experimentally validated configurations.

(2) The molecular docking carries intrinsic limitations, including approximations in scoring functions, limited sampling of protein and ligand conformational space, and incomplete treatment of solvent effects and entropic contributions. These factors can lead to both false-positive and false-negative binding mode assignments, and the predicted binding affinities should not be equated with experimentally determined values.

(3) In the absence of experimental structural validation by X-ray crystallography, NMR, or cryo-EM, the binding modes proposed here remain unconfirmed. Future work aimed at solving the structure of the protein-ligand complex will be essential to validate the contacts identified *in silico.*

(4) This study is based entirely on computational and *in vitro* experiments; therefore, it does not recapitulate the complex intracellular environment or the host immune response. The lack of macrophage infection assays and *in vivo* data limits the translational relevance of our findings. To address this, subsequent studies should incorporate cell-based infection models and relevant animal models to evaluate the functional consequences of the identified interactions in a physiologically meaningful context. Despite these limitations, the findings presented here provide a mechanistic framework that can guide the design of future experimental validation and help prioritize candidate compounds for further development.

In conclusion, our findings provide a detailed molecular mechanism for SerB2 function, positioning it as a validated and promising drug target. Developing small-molecule inhibitors that block its active site or substrate-binding pocket could re-sensitize Mycobacterium tuberculosis to the host’s innate immune defenses, offering a novel therapeutic strategy for combating tuberculosis.

## 4 Materials and methods

### 4.1 Protein constructs, expression, and purification

SerB2 constructs were prepared, expressed and purified (Supplementary Figures S2, S3) using previously reported methods (Mande et al., 2014; Shree et al., 2016; Haufroid et al., 2023). Briefly, 1 L of LB medium containing 100 μg/mL ampicillin was inoculated with 1% seed culture and grown overnight at 37°C with shaking at 180 rpm until an optical density (OD) at 600 nm (OD_600__nm_) of 0.6–0.8 was reached. Protein expression was induced by adding 0.5 mM IPTG (isopropyl-beta-D-thiogalactoside), and the culture was further grown for 8 h at 37°C, 120 rpm.

Cells were harvested, resuspended in buffer A (50 mM Tris-HCl, pH 8.0, 200 mM NaCl, 5 mM imidazole, and 12% glycerol), and lysed by sonication after adding 1 mM phenylmethylsulfonyl fluoride. Protein purification was performed using a Ni^2+^-IDA column (GE Healthcare) pre-equilibrated with buffer A, and protein was eluted using a linear imidazole gradient up to 1 M in buffer B (50 mM Tris-HCl, pH 8.0, 200 mM NaCl). Eluted fractions were analyzed by SDS-PAGE, pooled, and precipitated with 40% ammonium sulfate. The resulting pellet was resuspended in buffer C (50 mM Tris-HCl, pH 8.0, 50 mM NaCl, 5 mM beta-mercaptoethanol) and further purified by gel filtration using a Superdex S200 column (GE Healthcare) pre-equilibrated with buffer C. Protein was pooled and concentrated to 20 mg/mL using 10-kDa cutoff centricons.

### 4.2 Dynamic light scattering experiments

The oligomeric state of SerB2 was assessed using dynamic light scattering (DLS) with a Dynapro DLS instrument (Malvern Zetasizer, Malvern, United Kingdom). SerB2 was concentrated to approximately 2.1 mg/mL in buffer (150 mM NaCl, 20 mM Tris-HCl, pH 7.4) and then centrifuged at 18,000 rpm for 5 min at 4°C. The sample was loaded into a 1-cm path length cuvette, and data were acquired over 30 runs with an equilibration time of 120 s per run. The DLS data were analyzed using Zetasizer software (Version 6.20), which generated regularized histograms to determine the diameter, which was monitored throughout the analysis.

### 4.3 Structural model prediction and quality assessment

The structural model of SerB2 was predicted using AlphaFold2 (Wayment-Steele et al., 2023; Jumper et al., 2021) with the protein sequence (UniProt code O53289) as input. Molecular graphics were visualized using PyMOL v2.3.4.^2^

Structural validation was performed through geometric analysis. A Ramachandran plot, generated using PDBsum (Laskowski, 2022; de Beer et al., 2014; Laskowski, 2004, 2009; Laskowski et al., 2017), was used to assess the backbone dihedral angles (φ/ψ) and evaluate the stereochemical quality. This analysis identified favorable conformations and flagged energetically unfavorable residue orientations, providing quantitative validation of the predicted model’s reliability.

If the stereochemical stability of amino acids, as indicated by the proportion of residues located in the most favored region of the Ramachandran plot, falls below 94.8%, the following validation and refinement procedures will be undertaken. First, the electron density maps (2Fo-Fc and Fo-Fc) will be visually inspected for outlier residues to identify possible modeling errors, alternative conformations, or regions of poor density. Second, manual model adjustment will be performed using molecular graphics software such as Coot, with emphasis on backbone torsion angles, side-chain rotamers, and peptide bond flips. Third, restrained refinement will be repeated using programs such as REFMAC5 or PHENIX, applying increased weight to stereochemical restraints; simulated annealing or torsion-angle dynamics may be employed if necessary. Fourth, additional validation will be conducted using MolProbity, PROCHECK, or the wwPDB validation server to monitor improvements. Fifth, if the issue persists, overall data quality, including resolution, anisotropy and twinning, will be re-evaluated. Finally, if stereochemical quality remains below the threshold despite these efforts—whether due to inherent structural flexibility or low-resolution limitations—this limitation will be explicitly acknowledged in the manuscript, and alternative evidence supporting the structural conclusions will be emphasized.

### 4.4 Molecular docking

Molecular docking of O-phospho-L-serine to the SerB2 binding site was conducted using AutoDockTools 1.5.7 and AutoDock 4.2.6 (Huey et al., 2007; Morris et al., 2009; Trott and Olson, 2009; Forli et al., 2016). The Genetic Algorithm (GA) parameters were set to 1,000 runs, a population size of 200, with 30,000 generations and 3,000,000 evaluations. After the docking procedure, the results were analyzed, and the interactions between SerB2 and O-phospho-L-serine were visualized using PyMOL v2.3.4 (see text footnote 2). The binding energy was −5.92 kcaL/moL, which suggests that the docking results are reliable. Binding energy, typically expressed in kcal/mol, reflects the affinity of a ligand for its target protein, with more negative values indicating stronger binding and greater complex stability. This metric serves as a key indicator of functional modulation potential, guiding the identification of bioactive compounds for further investigation.

### 4.5 Enzymatic activity assay

Enzymatic activity of SerB2 and its mutants (Supplementary Table S1) was measured using a modified version of a previously described method (Haufroid et al., 2023; Mande et al., 2014; Shree et al., 2016; Liu et al., 2025b). Assays were performed in triplicate in 200 μL reactions containing 20 mM Tris-HCl (pH 7.5), 5 mM MgCl_2_, 1 mM DTT, 100 nM SerB2, and L-3-phosphoserine. Inhibition studies were conducted with the inhibitors AP3 (2-amino-3-phosphonopropionic acid), AP4 (2-amino-4-phosphonobutyric acid), GPC (L-alpha-glycerophosphorylcholine), and clofazimine.

Reactions were initiated by adding 100 nM SerB2 and incubated at 37°C for 30 min. The reactions were terminated by adding 200 μL of freshly prepared malachite green-ammonium molybdate dye reagent. After 1 min at room temperature, 10 μL of 34% citric acid was added to stop the reaction, and absorbance at 630 nm was measured using a Synergy4^^TM^ plate reader (BioTek).

### 4.6 MD (molecular dynamic) simulations

Molecular dynamics simulations were conducted using GROMACS 2022.3 (Abraham et al., 2015) with the AMBER99SB-ILDN force field (Lindorff-Larsen et al., 2010) and the TIP3P water model, based on the refined SerB2 model. Electrostatic interactions were computed using the PME method (Deserno and Holm, 1998a,b), with a 1.2 nm cutoff for both electrostatic and van der Waals interactions. The Verlet cutoff scheme was employed, and neighbor lists were updated every 10 steps (1.2 nm cutoff). Systems were maintained at 310 K and 1 bar using Nose-Hoover (Hoover and Holian, 1996) and Parrinello-Rahman (Parrinello and Rahman, 1981) thermostats (coupling constants: 0.4 and 0.1 ps, respectively), with periodic boundary conditions. Prior to production runs, the systems underwent energy minimization (steepest descent), followed by NVT (100 ps) and NPT (100 ps) equilibration. Production simulations were carried out for 200 ns with a 2 fs timestep. Trajectory analysis (RMSD/RMSF) was performed using GROMACS tools and visualized with xmgrace.

### 4.7 Bacterial growth curve

Bacterial growth kinetics were monitored in batch culture by measuring changes in optical density (OD) at 600 nm, which correlates with cell density. The resulting growth curve exhibited distinct phases corresponding to different physiological states of the bacterial population. For experimental conditions, cultures were grown to mid-logarithmic phase before treatment with 0 or 10 μM clofazimine.

Bacterial solutions were prepared in growth medium, inoculated to a starting concentration of 1.2 × 10^6^ CFU/mL, and incubated at 37°C with orbital shaking (150 rpm). Growth was assessed at 2, 4, and 6 days by measuring OD at 600 nm using a Synergy4^^TM^ plate reader (BioTek) in 96-well plates, with 100 μL aliquots taken for each measurement.

### 4.8 Expression levels of gene *SerB2* using RT-qPCR

To assess *SerB2* gene expression by real-time quantitative PCR (RT-qPCR), 1 mL of bacterial culture in late logarithmic phase was treated with 0 or 10 μM clofazimine at different time points (0, 2, 4, and 6 days). Total RNA was extracted using the TransZol RNA extraction kit (Invitrogen, Carlsbad, CA) according to the manufacturer’s protocol. cDNA was synthesized from RNA samples using the PrimeScript 1st Strand cDNA Synthesis Kit (Takara, Kyoto, Japan).

RT-qPCR was performed with PowerUp SYBR Green Master Mix (Applied Biosystems), and primers are listed in Supplementary Table S2. The qPCR analysis was carried out using the Applied Biosystems QuantStudio 5 instrument. Data were analyzed using the 2^−ΔΔCT^ method (Schmittgen and Livak, 2008; Livak and Schmittgen, 2001; Liu et al., 2025c), and relative expression was presented as a log_2_ ratio in a histogram. A ratio above zero indicated up-regulation, while a ratio below zero indicated down-regulation. The gyrase gene *gyrA* was used as a housekeeping reference for data normalization, with a positive control performed using *gyrA*.

### 4.9 Statistical analysis

All experiments were performed at least in triplicate, and data are presented as mean ± SD (standard deviation). The assumptions of normality and homogeneity of variances were verified using the Shapiro-Wilk test and Bartlett’s test, respectively. Differences among group means were evaluated by one-way ANOVA (one-way analysis of variance). When the overall ANOVA was significant, Tukey’s honestly significant difference (HSD) *post hoc* test was applied to identify which specific groups differed. Parametric tests were chosen because the data satisfied the normality and equal-variance assumptions. A *p* < 0.05 was considered statistically significant, and a *p* < 0.01 was considered highly significant. All statistical analyses were performed using SPSS 19.0, Origin 8.5, and Microsoft Excel 2013.

## Data Availability

The original contributions presented in the study are included in the article/Supplementary material, further inquiries can be directed to the corresponding authors.

## Footnotes

1
https://www.who.int/news-room/fact-sheets/detail/tuberculosis

2
https://www.pymol.org/2/
