# Supplementary material for: Molecular insights into the functional mechanism of phosphoserine phosphatase SerB2 in Mycobacterium tuberculosis
Source: Front Microbiol. 2026 Jul 2;17:1863671. doi: 10.3389/fmicb.2026.1863671 (PMC13372908; doi:10.3389/fmicb.2026.1863671)
Supplement: Supplementary file 1 [file Data_Sheet_1.docx]

**Supplementary Material**

**Figure S1.**


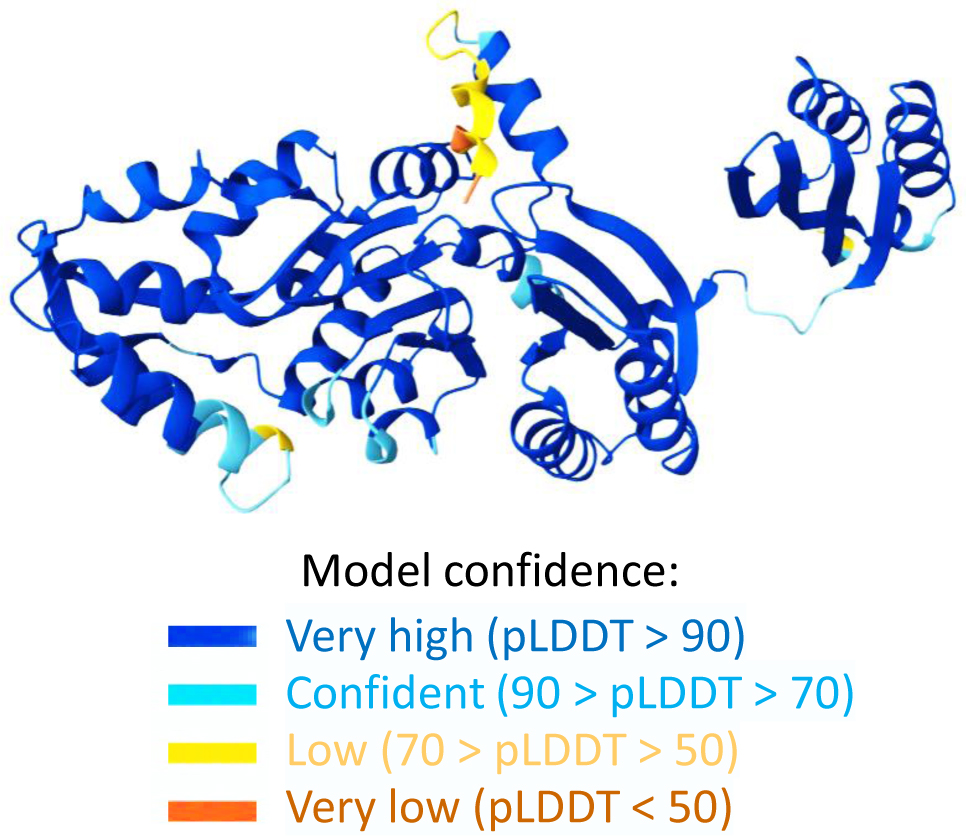


**Figure S1. The per-residue Local Distance Difference Test (pLDDT) was used to evaluate the confidence of the SerB2 structure predicted via AlphaFold2.** This metric spans a scale from 0 to 100, where higher values correspond to greater predicted local model accuracy.

**Figure S2.**

MPAKVSVLITVTGMDQPGVTSALFEVLAQHGVELLNVEQVVIRGRLTLGVLVSCPLDVADGTALRDDVAAAIHGVGLDVAIERSDDLPIIRQPSTHTIFVLGRPITAGAFSAVARGVAALGVNIDFIRGISDYPVTGLELRVSVPPGCVGPLQIALTKVAAEEHVDVAVEDYGLAWRTKRLIVFDVDSTLVQGEVIEMLAARAGAQGQVAAITEAAMRGELDFAESLQRRVATLAGLPATVIDDVAEQLELMPGARTTIRTLRRLGFRCGVVSGGFRRIIEPLARELMLDFVASNELEIVDGILTGRVVGPIVDRPGKAKALRDFASQYGVPMEQTVAVGDGANDIDMLGAAGLGIAFNAKPALREVADASLSHPYLDTVLFLLGVTRGEIEAADAGDCGVRRVEIPAD

**Figure S2. The amino acid sequence of SerB2 (UniProt code O53289).**

**Figure S3.**

ATGCCAGCTAAAGTCTCCGTACTGATTACCGTCACCGGTATGGACCAGCCGGGTGTTACTTCCGCACTGTTCGAAGTTCTGGCGCAGCACGGTGTAGAACTGCTGAACGTCGAACAGGTTGTAATTCGCGGTCGTCTGACCCTGGGCGTTCTGGTGTCTTGCCCGCTGGACGTAGCAGACGGTACCGCGCTGCGTGACGATGTTGCGGCTGCCATCCATGGCGTTGGCCTGGACGTTGCTATCGAACGCTCCGACGACCTGCCGATCATCCGCCAGCCTAGCACCCACACGATTTTCGTTCTGGGCCGCCCGATCACTGCAGGCGCGTTTAGCGCTGTTGCTCGTGGCGTCGCGGCACTGGGTGTGAACATCGATTTCATCCGTGGCATTAGCGACTACCCAGTGACCGGTCTGGAGCTGCGCGTGTCTGTACCGCCGGGTTGTGTCGGCCCGCTGCAGATCGCGCTGACCAAAGTTGCTGCGGAGGAACACGTCGATGTGGCGGTTGAGGACTACGGTCTGGCATGGCGTACCAAACGTCTGATCGTTTTCGATGTAGACAGCACCCTGGTGCAGGGCGAAGTGATCGAAATGCTGGCAGCGCGTGCAGGTGCCCAGGGCCAGGTAGCGGCTATCACTGAAGCGGCCATGCGTGGCGAGCTGGACTTCGCTGAATCTCTGCAACGTCGTGTCGCTACCCTGGCAGGTCTGCCTGCAACCGTTATCGATGACGTTGCGGAACAACTGGAACTGATGCCGGGCGCTCGTACGACGATTCGCACCCTGCGCCGTCTGGGCTTCCGTTGTGGCGTTGTCAGCGGCGGTTTCCGTCGCATTATCGAACCGCTGGCGCGCGAACTGATGCTGGATTTCGTTGCTTCTAACGAACTGGAAATCGTCGACGGCATTCTGACTGGTCGTGTAGTTGGTCCGATCGTTGATCGTCCGGGTAAAGCGAAAGCGCTGCGTGACTTCGCTTCCCAGTACGGTGTACCGATGGAACAGACTGTTGCTGTAGGTGATGGTGCGAACGATATCGATATGCTGGGTGCAGCCGGCCTGGGCATCGCTTTCAACGCGAAACCGGCACTGCGTGAAGTGGCGGATGCGAGCCTGTCCCACCCGTATCTGGACACGGTTCTGTTCCTGCTGGGTGTTACCCGTGGTGAAATTGAAGCAGCGGATGCGGGCGATTGTGGCGTTCGTCGTGTTGAAATCCCGGCTGAC

**Figure S3. Gene sequence of *SerB2* after codon optimization**

**Table S1.**

**Table S1. Primers used for generating site-directed mutants of *SerB2***

| Primers | Primer sequence (5’-3’) |
| --- | --- |
| D185A (F) | GATCGTTTTC**gca**GTAGACAGCACCCTGGTGC |
| D185A (R) | GCTGTCTAC**tgc**GAAAACGATCAGACGTTTGGTAC |
| D187A (F) | TTCGATGTA**gca**AGCACCCTGGTGCAGGG |
| D187A (R) | CAGGGTGCT**tgc**TACATCGAAAACGATCAGACG |
| E194A (F) | GTGCAGGGC**gca**GTGATCGAAATGCTGGC |
| E194A (R) | CGATCAC**tgc**GCCCTGCACCAGGG |
| S226A (F) | CGCTGAA**gca**CTGCAACGTCGTGTCGCTACC |
| S226A (R) | CGTTGCAG**tgc**TTCAGCGAAGTCCAGCTCGC |
| R230A (F) | CTGCAACGT**gca**GTCGCTACCCTGGC |
| R230A (R) | GGTAGCGAC**tgc**ACGTTGCAGAGATTCAGCGAAG |
| K318A (F) | GATCGTCCGGGT**gca**GCGAAAGCGCTGCGTGAC |
| K318A (R) | GCTTTCGC**tgc**ACCCGGACGATCAACGATCGGAC |
| D341A (F) | CTGTAGGT**gca**GGTGCGAACGATATCGATATGCTGGG |
| D341A (R) | GTTCGCACC**tgc**ACCTACAGCAACAGTCTGTTCCATC |
| D345A (F) | GTGCGAAC**gca**ATCGATATGCTGGGTGCAGCC |
| D345A (R) | CATATCGAT**tgc**GTTCGCACCATCACCTACAGCAACAGTCTG |

Note: Mutagenic regions in the sequence are underlined and displayed in bold.

**Table S2.**

**Table S2. Primers used for RT-qPCR**

| Primers | Primer sequence (5’-3’) |
| --- | --- |
| *gyrA* (F) | atgacagacacgacgttgccgcc |
| *gyrA* (R) | ttaattgcccgtctggtctgcgcc |
| *serB2* (F) | atgccagccaaggtgtcggtg |
| *serB2* (R) | ctagtcggccgggatttcgacg |
